# Similarity-aware VAE with wavelet-convolutional 1D-CNN for rolling bearing fault diagnosis

**DOI:** 10.1371/journal.pone.0338388

**Published:** 2026-01-05

**Authors:** Wei Xiong, Na Xiao, Ruili Wang

**Affiliations:** Faculty of Engineering, Huanghe Science and Technology College, Zhengzhou City, China; The Hong Kong Polytechnic University, CHINA

## Abstract

To address the uneven distribution of fault categories in data sets for deep learning-based fault diagnosis, we propose a fault diagnosis framework combining an improved Variational Autoencoder (Similarity-Aware VAE) with a Wavelet-Convolutional 1D-CNN. The Similarity-Aware VAE employs a novel similarity loss function for data augmentation, measuring feature distances in high-dimensional space while automatically adjusting training parameters and weights through an enhanced attention mechanism to balance the dataset.The Wavelet-Convolutional 1D-CNN replaces the first convolutional layer of CNN with a Wavelet-Convolutional layer based on continuous wavelet transform, enabling multi-scale feature extraction for fault data analysis. Experimental validation using public datasets demonstrates that this method effectively enhances data quality while maintaining robust diagnostic performance, offering practical implications for industrial fault diagnosis.

## 1 Introduction

Gears, bearings, and other components are indispensable parts of rotating machinery, widely used in wind power, automotive, aviation, and other mechanical equipment [1]. The health of rotating parts is crucial to the overall safety and reliability of the mechanical system. According to statistics, bearing failures account for about 30% of faults in rotating machinery, while gear failures account for about 60%.

Traditional signal processing diagnostic methods, due to the need for complex signal analysis algorithms to extract features and the heavy reliance on expert experience in feature analysis and fault diagnosis, have been significantly limited in application [2].

In the process of using these classic methods, it is usually necessary to manually extract and analyze signals [3].

For complex equipment structures and multi-classification problems, relying solely on traditional pattern recognition methods often fails to accurately represent the mapping relationships between data [4].

Deep learning, through deep nonlinear structures to learn the intrinsic laws of data and construct deep learning methods that map between samples and results, has begun to attract attention in the field of fault diagnosis [5]. Wu [6] et al. utilized one-dimensional (1-DCNN) convolutional neural networks to directly learn features from raw vibration signals, followed by fault diagnosis. Hoang [7] et al. proposed a bearing fault diagnosis method based on the deep structure of convolutional neural networks, which exhibits high accuracy and robustness in noisy environments. Shao [8] et al. introduced a multi-signal fault diagnosis method based on DL, where the deep model can automatically learn and select appropriate features, aiding in accurate fault diagnosis. Compared to single-signal input, multi-signal models offer more precise and stable performance, effectively overcoming overfitting issues to some extent. Tang [9] et al. summarized and discussed DL-based fault diagnosis methods for rotating machinery, outlining the challenges faced by modern intelligent fault diagnosis and potential future research directions. However, in engineering scenarios, machines are mostly in normal operating conditions with few faults occurring, resulting in limited and unevenly distributed data; fault simulation experiments in laboratories require the artificial creation of faults and the establishment of fault test benches, which are costly [10].

This paper addresses the issue of uneven data distribution in rolling bearing fault diagnosis and proposes a method based on SA-VAE and WaveNet for rolling bearing fault diagnosis. The method introduces a similarity loss function in VAE, measures the Wasserstein distance between high-dimensional data features, and uses an improved adaptive parameter-adjustable attention mechanism to focus on learning important features for data reconstruction. It achieves rolling bearing fault diagnosis through wavelet convolution networks.

## 2 fundamental theory

### 2.1 Analysis of common failure types and mechanisms of rolling bearings

#### 2.1.1 Common bearing failure types.

Based on the structure of rolling bearings, they consist of an inner ring, outer ring, rolling elements, and cage. Various bearing failures in rotating machinery are the primary causes of abnormal vibration responses in mechanical equipment [11]. The normal service life of rolling bearings in operation is determined by material failure and wear on the bearing’s moving surfaces [12]. Common failure modes of rolling bearings include: 1) Fatigue failure; 2) Abrasive failure; 3) Plastic deformation; 4) Corrosion failure; 5) Fracture failure. Bearing fractures or breakage may occur due to improper assembly, usage, maintenance, or inadequate lubrication, resulting in fracture failure.

In kinematics, normal rolling bearings maintain fixed natural frequencies during operation, which are determined by their dimensional parameters. When faulty bearings operate for extended periods or develop failure points due to external factors, they exhibit periodic vibrations during rotation. This causes the natural frequency of the faulty bearing to deviate from that of a healthy one. If the measured frequency significantly differs from the theoretical frequency, the bearing is considered defective.

The rolling elements and raceways are treated as pure rolling. Assuming a fixed contact angle α during motion, with Z rolling elements, ${\text{f}}_{\text{i}}\;\;$rotation frequency of the inner ring, D bearing pitch diameter (radius of the circle containing rolling elements), and d rolling element diameter, the characteristic failure frequencies for different component locations are derived as follows.

The fault frequency of the outer ring is:


$$f_{Fo}=\frac{Z}{\text{2}}f_{i}\left(1-\frac{d}{D}\cos a\right)$$ (1)

The fault frequency of the inner circle is:


$$f_{Fi}=\frac{Z}{\text{2}}f_{i}\left(1+\frac{d}{D}\cos a\right)$$ (2)

The fault frequency characteristics of rolling body are as follows:


$$f_{Fb}=\frac{D}{2d}f_{i}\left(1-{\left(\frac{d}{D}\cos a\right)}^{2}\right)$$ (3)

The fault frequency characteristics of the support are as follows:


$$f_{Fc}=\frac{f_{i}}{\text{2}}\left(1-\frac{d}{D}\cos a\right)$$ (4)

#### 2.1.2 Continuous wavelet transform CWT.

The continuous wavelet transform (Continuous wavelet transform, CWT) based on inner products is a well-known signal processing method that exhibits excellent adaptability for time-varying and non-stationary signals. It provides good temporal resolution for the high-frequency components of the signal and good frequency resolution for the low-frequency components [13]. Applying the preprocessed vibration signals to the continuous wavelet transform allows for local analysis of the signal in both time and frequency domains, providing more detailed information. Compared to the short-time Fourier transform with fixed window functions, the continuous wavelet transform can use adjustable window functions [14].

For any signal, its x(t)continuous wavelet transform is defined as:


$$CWT_{f}\left(u,s\right)=\left\langle g,\psi_{u,s}\left(t\right)\right\rangle =\frac{\text{1}}{\sqrt{s}}\int x\left(t\right)\psi *\left(\frac{t-u}{s}\right)dt$$ (5)

Among them, the conjugate $\psi^{*}(∙)\;$complex is represented as the mother wavelet. The shape and displacement of the wavelet function are determined by parameters and. The formula of the wavelet basis function is as follows:


$$\psi_{u,s}\left(t\right)=\frac{\text{1}}{\sqrt{s}}\psi \left(\frac{t-u}{s}\right)$$ (6)

**Fig 1:**
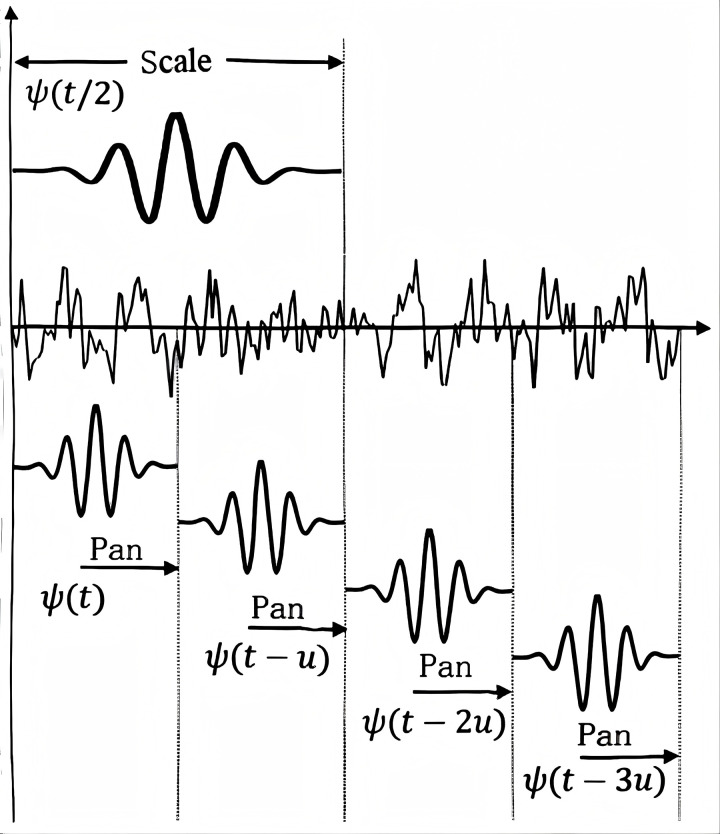
Signal matching of CWT.

Among them, ψ is the mother wavelet function; u is the translation parameter;s is the scale parameter;t is time. Parameters can be used to scale signals, as shown in Fig 1. Wavelet basis functions can quickly characterize signal features. Selecting appropriate wavelet basis functions and scale parameters can provide a more detailed representation of the signal’s components.

### 2.2 Attention mechanism

Attention Mechanism is a mechanism used to increase the focus on specific parts of input information. Introducing the Attention mechanism allows the model to focus on important features from a large amount of information and learn comprehensively. For signal features, the Attention mechanism generates weight coefficients, assigning greater weights to more important features and smaller weights to less important ones, ultimately summing up the weighted values and extracting more significant features [15]. The expression for the attention mechanism is:


$$Attention\left(Q,K,V\right)=soft\max\left(\frac{QK^{T}}{\sqrt{d_{k}}}\right)V$$ (7)

The Attention is primarily composed of three parts: Q (Query), K (Key), and V (Value). Q, K, and V are vectors or matrices obtained from the linear transformation of input sequences. The computation in the Attention mechanism module mainly consists of three steps: the first step is to compute the attention score between Query and Key using a similarity function; the second step is to normalize the attention scores using Softmax to obtain weight coefficients; the third step is to perform a weighted sum of Values based on the distribution of these weight coefficients.

### 2.3 variational autoencoder VAE

Variational autoencoders are a type of data generation model in the field of deep learning. The primary method of VAE is to extract the hidden features of the learning object and generate new data samples from them. This model can learn potential attributes from the probability distribution in the latent variable space and construct new elements. VAE consists of two parts: the encoder Encoder and the decoder Decoder.As shown in the figure, the input data $X=\left\{X_{1},X_{2},X_{3},...,X_{N}\right\}$ is fed into the encoder, which calculates its mean and variance, then extracts features from the latent variables and samples to obtain. The decoder $Z=\left\{Z_{1},Z_{2},Z_{3},...,Z_{N}\right\}$ then generates new data based on this $Z=\left\{Z_{1},Z_{2},Z_{3},...,Z_{N}\right\}$. In this process, the sampled data features Z from the latent variable space come from X and both follow a normal distribution.

The specific formula is:


P(Z)=∑P(Z|X)P(X) (8)

The VAE model can be expressed as:


$$P\left(X\right)=\int_{Z}P\left(x|z\right)P\left(z\right)dz$$ (9)

By using the variational inference method, the variational lower bound (ELBO) is optimized to maximize the marginal likelihood of the data. ELBO includes reconstruction error and KL divergence loss:


$$ELBO=Loss_{Re\mathrm{\backslash nolimits}c}+Loss_{KI}=E_{q\left(Z|X\right)}\left[\log p\left(x|z\right)\right]-D_{KL}\left(q\left(z|x\right)|p\left(z\right)\right)$$ (10)

**Fig 2:**
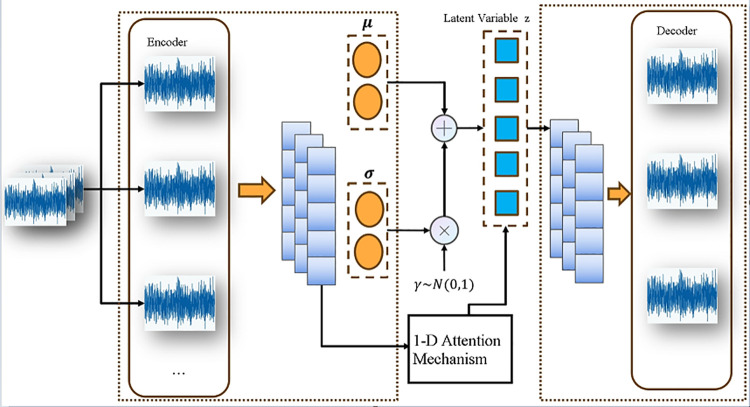
VAE structure.

Variational autoencoders can be defined as an autoencoder whose training is regularized to avoid overfitting and ensure that the potential space has good properties to support the generation process.As shown in Fig 2, the application architecture of variational autoencoders in data processing, with the core being the realization of feature learning through the ‘encoding-latent variable generation-decoding’ process.

## 3. Rolling bearing fault diagnosis method based on SA-VAE data enhancement

### 3.1 Data generation model based on similarity loss is proposed

In order to maintain the local structure of data in the potential space, a new loss function based on similarity is proposed to compare the distance between data distribution in the low-dimensional potential space. The specific form of the loss function in the VAE framework is [16]:


$$Loss_{Similarity}=\frac{\text{1}}{N}\sum {\mathrm{\backslash nolimits}}_{i=1}^{N}W_{i}\log\left(\frac{\exp\left(Q_{i}\right)}{Q_{i}}\right)$$ (11)

Among them, it represents ${\text{W}}_{\text{i}}{\text{Q}}_{\text{i}}$the distance between data points in the category space and is the distance of data distribution, which is a variant of one-dimensional Wasserstein distance.

Wasserstein distance is a mathematical method used to measure the difference between two probability distributions. Compared with traditional distance metrics like Euclidean distance, Wasserstein distance takes into account the similarity between distributions and the relationship between geometric distances, making it more suitable for describing the similarity of high-dimensional datasets. Its advantage over KL divergence and JS divergence lies in its ability to reflect the proximity of two distributions even when their support sets do not overlap or only partially overlap, effectively representing the distance between different fault categories in fault diagnosis. Using a similarity loss function can make the sample features in the original latent variable space more concentrated, resulting in data that is closer to the original data.

### 3.2 Improved attention mechanism

In order to make the model applicable to one-dimensional signals, this study adopts a one-dimensional signal attention mechanism, so that the model can pay attention to the main aspects of features [17]. In addition, different from the conventional activation functions such as ReLU, this paper adopts ACON activation function, the specific formula is as follows:


$$f_{ACON}=\left(p_{1}x-p_{2}x\right)sigmoid\left[\beta \left(p_{1}x-p_{2}x\right)\right]+p_{2}x$$ (12)

**Fig 3:**
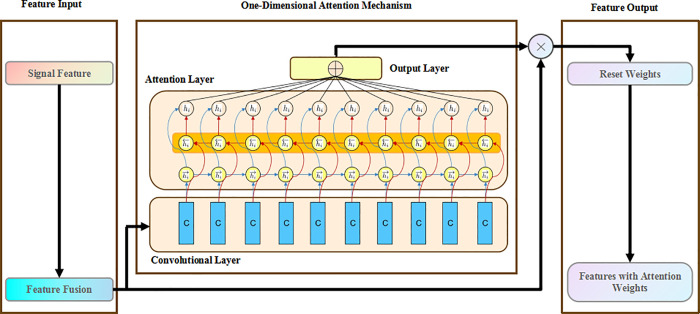
One-dimensional attention mechanism proposed.

Among them, x is the input, ${\text{p}}_{\text{1}}$and ${\text{p}}_{\text{2}}\;$are the parameter that can be adaptively adjusted to p1 and p2, and β is a gating parameter produced by a lightweight auxiliary network. The activation function can be adaptively adjusted to ${\text{p}}_{\text{1}}$ and ${\text{p}}_{\text{2}}$ through back propagation.As shown in Fig 3, a feature processing architecture combined with a one-dimensional attention mechanism. By performing convolutional extraction and attention weighting on the input signal features, it outputs features that highlight key information to improve the accuracy and robustness of subsequent tasks.

The attention mechanism can be viewed as a cost-sensitive learning method. The addition of new activation functions has a regularization effect on features, making the proposed attention mechanism more conducive to improving the model’s generalization [18]. However, using only the SA-VAE model cannot achieve fault diagnosis, hence a rolling bearing fault diagnosis model based on SA-VAE and WaveNet is proposed.

### 3.3 Rolling bearing fault diagnosis method based on SA-VAE and Wavelet-Convolutional 1D-CNN

**Fig 4:**
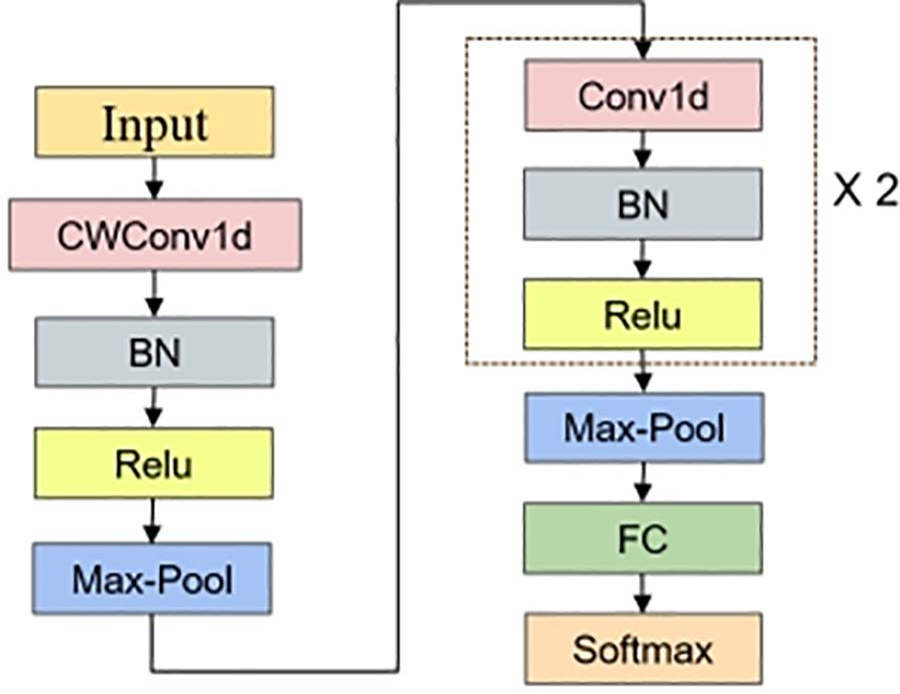
CWConv-1D-CNN structure.

**Fig 5:**
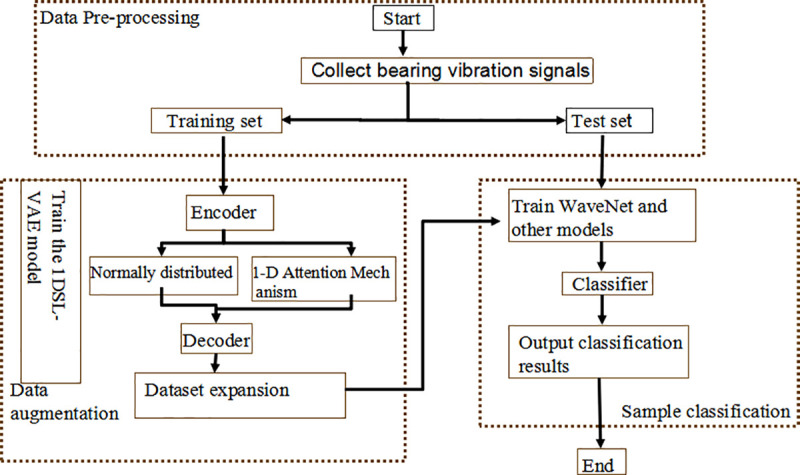
Flow chart of SA-VAE and Wavelet-Convolutional 1D-CNN fault diagnosis algorithm.

The data generation model based on SA-VAE is used to extract the signal features, and the data features are input into the wavelet convolutional neural network model WaveNet to form a learning model with data enhancement and fault diagnosis. The algorithm process is shown in Fig 4 and Fig 5.

The method is divided into two stages. The first stage involves training the data-enhanced SA-VAE model. The dataset is fed into the SA-VAE model, which learns the data characteristics of different fault categories and measures the distance between the data distributions of these categories [19]. Meanwhile, the attention mechanism ensures that the model focuses on important features, thereby generating higher-quality data samples and achieving data enhancement before pre-training for fault diagnosis.

The second stage involves the training of the fault diagnosis model WaveNet. A portion of the generated sample data is blended with real-world samples to form an augmented dataset. When fed into the fault diagnosis model, the data first undergoes a wavelet convolution layer based on wavelet transform principles. Compared to conventional CNN convolution layers, this wavelet convolution layer demonstrates superior vibration signal feature extraction capabilities and enhanced fault diagnosis performance.

The execution steps of SA-VAE and Wavelet-Convolutional 1D-CNN fault diagnosis algorithm are based on.

1 Train the SA-VAE model, repeat step 1) -step 2), until the reconstructed data σ losserror is less than;
  1) Sample n segments of the signal from the training data ${\text{w}}_{\text{1}}\sim {\text{w}}_{\text{n}-\text{1}}$ set.
  2) Set the learning rate, calculate the loss function every cycle, and perform ${\text{Loss}}_{\text{Similarity}}$stochastic gradient descent to optimize the network encoder parameters δ and decoder parameters θ.
  3) The optimal δ、θ parameters, the generated data and the annotated data are input into the subsequent fault diagnosis model.
2 The trained SA-VAE data is connected to the fault diagnosis model WaveNet.
  1) Collect samples from generated data$\;\;\;\tau_{\text{1}}asciitilde\tau_{\text{n}-\text{1}}$.
  2) Gradient descent is performed on each sample, and weights are updated according ${\text{W}}_{\text{i}}=\text{SGD}({\text{L}}_{\tau_{\text{i}}})$to back propagation.
  3) The weighted average of the updated weights of all samples $\frac{\text{1}}{\text{k}}\sum_{\text{i}=\text{1}}^{\text{n}}{\text{W}}_{\text{i}}$ is taken.
3 Verify the model on the test set and obtain the performance indicators of the model.

## 4 Experimental analysis

### 4.1 Data description

**Fig 6:**
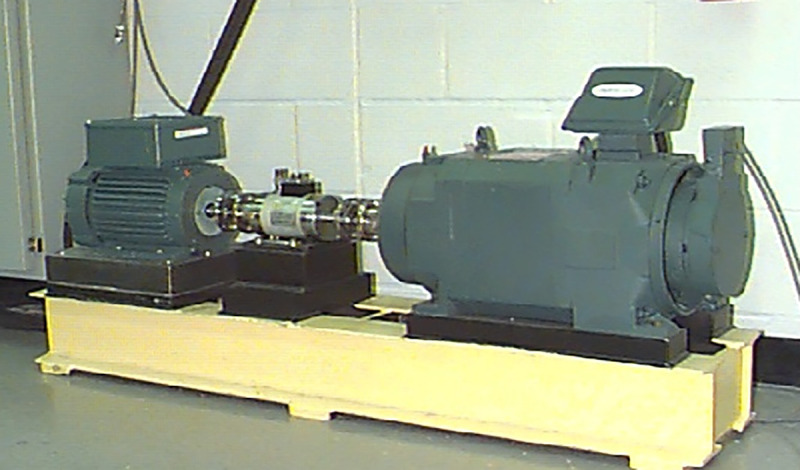
CWRU vibration test bench.

**Fig 7:**
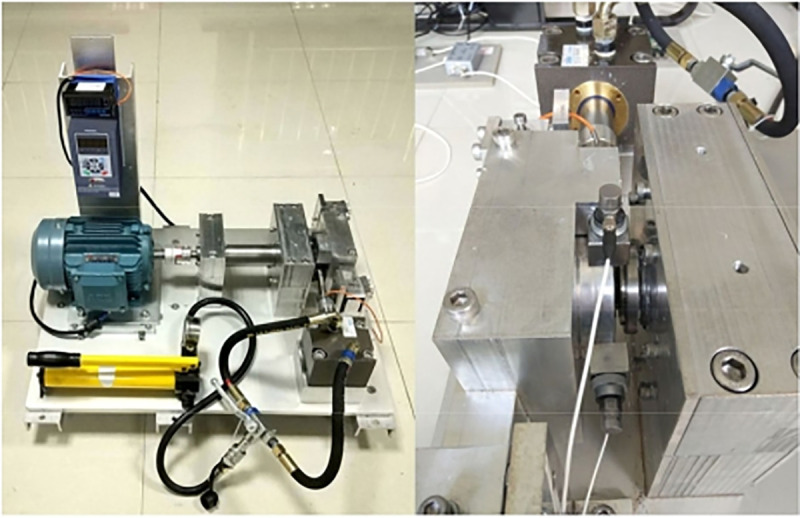
XJTU-SY vibration test bench.

The CWRU dataset and the XJTU-SY dataset are currently the most widely used standard bearing fault diagnosis datasets. The test bench for the CWRU dataset consists of a 2-horsepower motor, a power meter, and a torque sensor, designed for different motor loads. Single-point faults of varying severity are created at three different locations on the tested bearings. The accelerometer is fixed to the drive end and fan end bearing housings; the experimental platform for the XJTU-SY dataset comprises an AC motor, a motor speed controller, a shaft, support bearings, a hydraulic loading system, and the test bearing. The adjustable conditions of the test platform mainly include radial force and speed, with the radial force generated by the hydraulic loading system acting on the bearing housing of the test bearing, and the speed set and adjusted by the motor speed controller of the AC motor.The vibration test bench for CWRU and XJTU-SY is shown in Figs 6 and 7.

The CWRU bearing dataset includes vibration data for four different fault modes: normal condition, inner ring fault, outer ring fault, and rolling element fault. The dataset provides experimental data under various operating conditions, including different speeds, loads, and working times. Each fault mode has multiple samples under different operating conditions, with various classification forms based on different constraint conditions; the XCTU-SY dataset experiment was designed with 3 types of operating conditions, each type consisting of 5 bearings. The sampling frequency is 25.6kHz. The sampling interval is 1 min, and each sample lasts 1.28s. Each signal is divided into 1024 non-overlapping points, with a training-to-test ratio of 4:1, resulting in 512 training samples and 128 test samples. This study uses data from five fault conditions at a speed of 35 Hz and a load of 12 kN.

The parameters of the CWRU dataset are shown in Table 1 and those of the XJTU-SY Bearing Dataset are shown in Table 2.

**Table 1:** CWRU Data set parameters.

Data description	Fault Location	Fault Location	Label
For 12k Drive End Bearing Fault Data Motor Speed(0HP):1797 rpm	Inner Raceway	.007	0
	Inner Raceway	.014	1
	Outer Raceway	.007	2
	Outer Raceway	.014	3
	Ball	.007	4

**Table 2:** Description of XJTU‑SY Bearing Dataset.

Operation condition	File	Lifetime	Fault element
Speed: 35 Hz Load: 12kN	Bearing 1	8 h 11 min	Inner race
	Bearing 2	2 h 41 min	Outer race
	Bearing 3	8 h 53 min	Ball
	Bearing 4	42 min	Outer race
	Bearing 5	5 h 39 min	Outer race

### 4.2 Parameter settings

For the parameter and optimization method setting in the model training process, Adam optimizer is adopted in this paper, with the learning rate set to 0.0001, batch size of 64 and 50 epochs. In order to eliminate the randomness of the experiment, each group of experiments is conducted five times.

### 4.3 Analysis and conclusion

**Fig 8:**
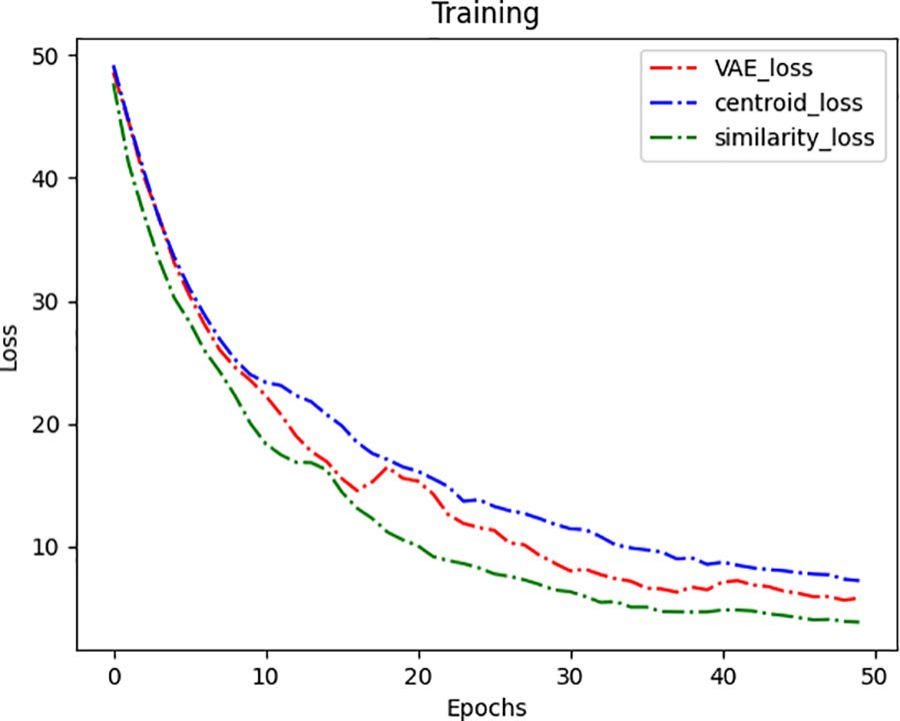
Gradient variation of different loss functions in SA-VAE.

This paper first validates the SA-VAE module. Since the proposed similarity loss function is a key component, the change in the model’s loss gradient is an important indicator. The training results of VAE models using different loss functions are compared, including the traditional VAE loss function (reconstruction loss and KL divergence), centroid loss, and similarity loss, as shown in Fig 8.

**Fig 9:**
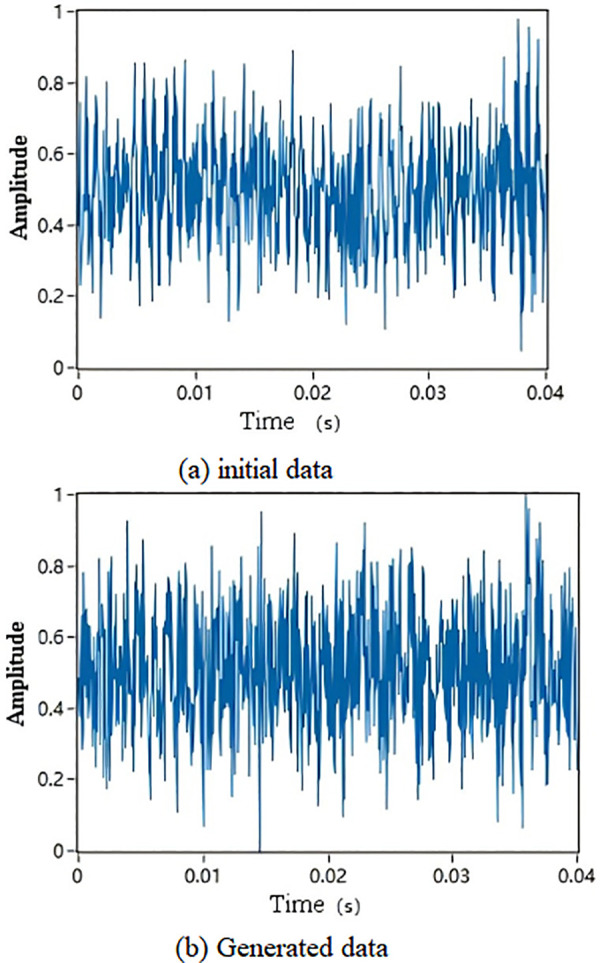
SA-VAE generated data (sampling rate 25.6kHz, sample length 1024).

It can be observed that in 50 rounds of iterative training, the VAE model based on centroid loss converges the slowest. This is because the centroid loss causes features to become overly concentrated, leading to the loss of some features during training. The loss gradient of the traditional VAE loss function converges more slowly compared to the other two loss functions, with an unstable curve. In contrast, The proposed similarity loss function achieves the lowest loss during training and shows a more stable performance compared to VAE. This is because the similarity loss function allows the model to measure the distance between major features in high-dimensional space during training. Unlike centroid loss, it does not overly focus on central features, and the attention mechanism makes the model pay more attention to important features, thus achieving rapid and stable convergence. Fig 9 shows an example of data generated by SA-VAE.

**Fig 10:**
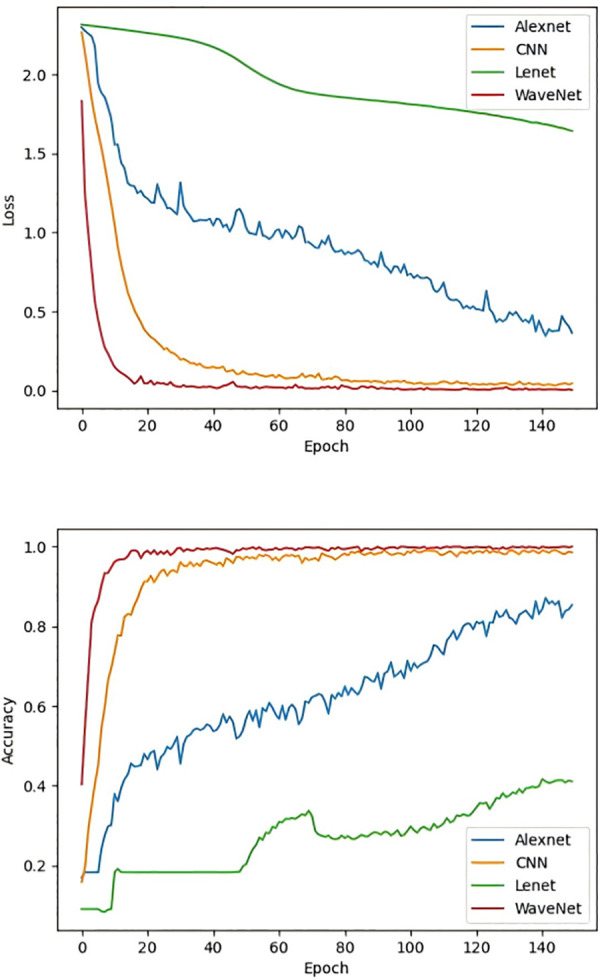
Training results of SA-VAE model.

After verifying the effectiveness of the SA-VAE module, the enhanced data was input into the fault diagnosis module. In this paper, WaveNet model was mainly adopted, and several other conventional fault diagnosis models, such as AlexNet, CNN, LeNet, etc., were also compared. The results are shown in Fig 10.

From the results, it can be seen that as the training cycle increases, CNN and WaveNet maintain good learning progress during the training process. Around 30 cycles, the training accuracy of CNN and WaveNet reaches its maximum and remains stable. According to statistics, when the model stabilizes, the average accuracy of CNN is 98.85%, and that of WaveNet is 99.98%. Moreover, The WaveNet training curve shows overall stability, with both loss and accuracy reaching optimal values in earlier epochs, indicating that using wavelet convolution layers to replace traditional convolution layers in WaveNet can more effectively extract signal features. On the other hand, the models of AlexNet and LeNet have poor stability, with significant fluctuations in accuracy and loss functions, suggesting that AlexNet and LeNet have weak learning capabilities for one-dimensional signal features. In summary, the data generated by the SA-VAE algorithm can be effectively used in traditional deep learning models, and the effectiveness of SA-VAE has been proven.

**Fig 11:**
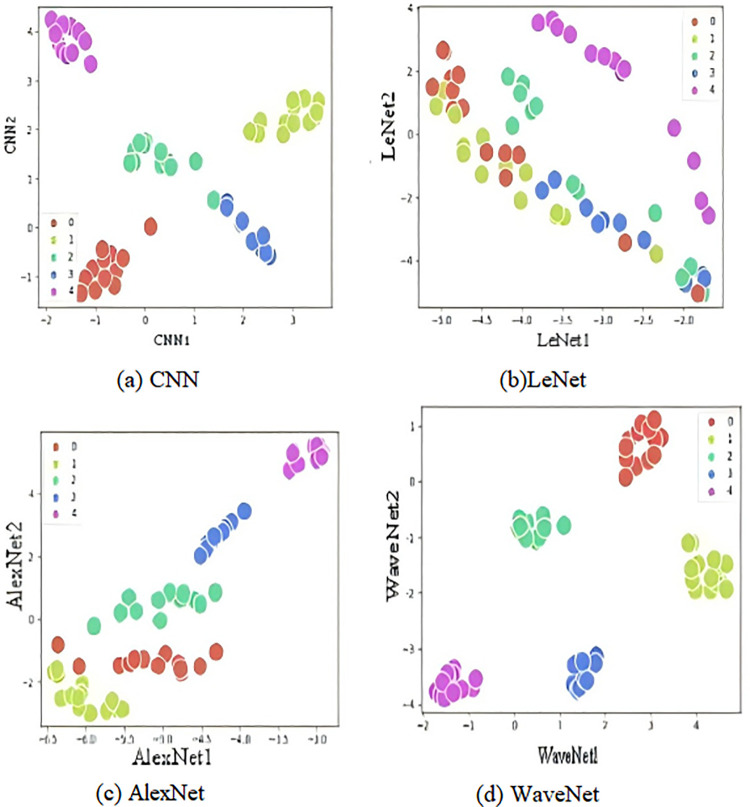
K-Mean clustering results: (a) CNN; (b) LeNet; (c) AlexNet; (d) WaveNet.

To verify the fault diagnosis effectiveness of the model, this study used the k-means clustering algorithm to classify the features output by the model. The k-means algorithm is an unsupervised clustering method based on data partitioning; it does not require labeling of the training data. It only needs to preset the number of clusters K, after which the data will be automatically divided into K categories using the k-means algorithm. The k-means algorithm is fast, simple, and has good clustering performance with nearly linear time complexity, requiring only the adjustment of the cluster number K. For the model output, cluster analysis was conducted, and each model randomly outputs a set of training data at the end of each training round, which was then classified using the k-means clustering algorithm to analyze the classification performance of the model. The specific results are shown in Fig 11.

**Fig 12:**
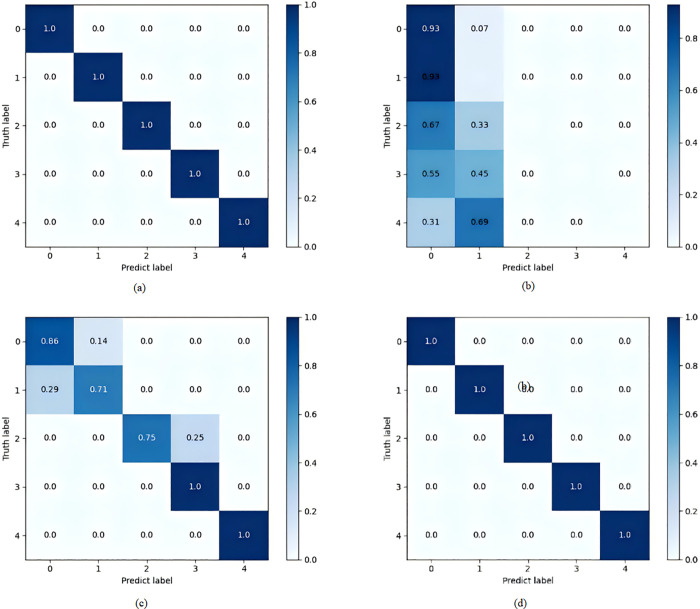
Confusion matrix of training results: (a) CNN; (b) LeNet; (c) AlexNet; (d) WaveNet.

It can be seen that after multiple rounds of iterative training, the models with more pronounced clustering effects are CNN and WaveNet. Among them, CNN has better clustering performance, while WaveNet shows the most significant clustering, indicating that the features retained from the signal data after passing through WaveNet are more detailed and more distinct. Although LeNet can perform simple classification, the distances between classes are too close to make clear distinctions; AlexNet cannot effectively classify. Therefore, through comparison, it is found that WaveNet has higher fault diagnosis performance. The confusion matrices in the training results of each model also fully illustrate this point, as shown in Fig 12.

The diagnostic performance of the two data sets is shown in Table 3.

**Table 3:** Model training results.

data set	model	Average accuracy (%)
XJTU‑SY	CNN	98.70
	LeNet	72.14
	AlexNet	93.49
	WaveNet	99.93
CWRU	CNN	98.85
	LeNet	68.97
	AlexNet	96.17
	WaveNet	99.98

Through the verification of two different data sets, both CNN and WaveNet show good performance, but WaveNet has higher accuracy, better convergence and classification effect, indicating that it is more suitable for one-dimensional vibration signal fault diagnosis, and also proves that SA-VAE algorithm can be effectively applied to fault diagnosis.

## 5 Conclusion

In response to the uneven distribution of different fault categories in data sets when deep learning is applied to fault diagnosis, a fault diagnosis framework based on an improved variational autoencoder SA-VAE and wavelet convolutional network WaveNet is proposed. SA-VAE is used for data augmentation to generate fault data to balance the dataset, while WaveNet performs feature learning on the fault data and conducts fault diagnosis. The method is validated using public datasets. Experimental results show that compared with other models, the proposed model exhibits better robustness and diagnostic performance. The main contributions of this paper are as follows:

(1) A SA-VAE data enhancement method based on similarity loss function is proposed to measure the distance of vibration data in high-dimensional feature space. The data generated by this method retains more features than the data generated by traditional VAE method.
(2) An improved attention mechanism is proposed. Compared with the traditional attention mechanism, it is more suitable for one-dimensional signals. The improved attention mechanism can automatically update parameters, which increases the generalization ability of the model.
(3) The proposed fault diagnosis method based on SA-VAE and WaveNet is verified by using different data sets. Experiments show that the method can enhance data to solve the problem of data imbalance in actual working conditions.

## References

[1] Zhang J, Ma J. Fault prediction of rolling bearings based on deep learning. Modern Electronic Technology. 2024;47(24):120–30.

[2] Zhang K, Cao ZH, Liu PF. Feature extraction of rolling bearing fault impact based on secondary CEEMDAN and CCJC [J]. Noise and Vibration Control, 2025,45(01):112–8+247.

[3] Zhang XL, Fan PF, Li XY. Fault diagnosis of rolling bearing order based on frequency transformation ridge line. Noise and Vibration Control. 2025;45(01):139–45.

[4] Liang XY, Hu YL, Ma XY. Rolling bearing fault diagnosis based on feature mode decomposition and multi-scale fuzzy dispersion entropy. Science and Technology & Engineering. 2025;25(01):176–85.

[5] Huang Y, Hu X, Wang H, He Y, Cao J. OAIFAN: A Noise-Robust Discriminative Feature Unification Framework for Cross-Speed Fault Transfer Diagnosis. IEEE Trans Instrum Meas. 2025;74:1–18. doi: 10.1109/tim.2025.3577843

[6] Wu ZC, Jiang PC. Intelligent fault diagnosis of rotating machinery based on one-dimensional convolutional neural network. Comput Ind. 2019;108(1):53–61.

[7] Hoang D, Kang H. Rolling element bearing fault diagnosis using convolutional neural network and vibration image. Cognit Syst Res. 2019;53(1):42–50.

[8] Shao S, Yan R, Lu Y, Wang P, Gao RX. DCNN-Based Multi-Signal Induction Motor Fault Diagnosis. IEEE Trans Instrum Meas. 2020;69(6):2658–69. doi: 10.1109/tim.2019.2925247

[9] Tang S, Yuan S, Zhu Y. Deep learning-based intelligent fault diagnosis methods toward rotating machinery. IEEE Access. 2020;8:9335–46. doi: 10.1109/access.2019.2963092

[10] Yang Z, Mao R, Ye L, Liu Y, Hu X, Li Y. VSC-ACGAN: bearing fault diagnosis model applied to imbalanced samples. Meas Sci Technol. 2025;36(3):036212. doi: 10.1088/1361-6501/adb872

[11] Chen F, Zhao Z, Hu X, Liu D, Kang Z, Ma Z, et al. Enhancing the safety of hydroelectric power generation systems: an intelligent identification of axis orbits based on a nonlinear dynamics method. Energy. 2025;324:135864. doi: 10.1016/j.energy.2025.135864

[12] Zhang ZG, Jiang Q, Zhan YB. A small sample rolling bearing fault classification method based on VAE-GAN data enhancement algorithm. Atomic Energy Science and Technology. 2023;57(S1):228–37.

[13] Kankar PK, Sharma SC, Harsha SP. Rolling element bearing fault diagnosis using wavelet transform. Neurocomputing. 2011;74(10):1638–45. 10.1016/j.neucom.2011.01.021

[14] Kankar PK, Sharma SC, Harsha SP. Fault diagnosis of ball bearings using continuous wavelet transform. Applied Soft Computing. 2011;11(2):2300–12. doi: 10.1016/j.asoc.2010.08.011

[15] Wang B, Ning Y, Zhang YH. A novel fault diagnosis scheme for rolling bearing based on symbolic aggregate approximation and convolutional neural network with channel attention. Meas Sci Technol. 2022;33(1):015016.

[16] Ma H. Achieving deep clustering through the use of variational autoencoders and similarity-based loss. Math Biosci Eng. 2022;19(10):10344–60. 10.3934/mbe.2022484 36031997

[17] Zhang X, He C, Lu Y, Chen B, Zhu L, Zhang L. Fault diagnosis for small samples based on attention mechanism. Measurement. 2022;187:110242. doi: 10.1016/j.measurement.2021.110242

[18] Mo J, Zhang Z. Attention multiscale convolutional neural network prediction of remaining service life of bearings. Modern Manufacturing Engineering. 2023;2023(8):148–54.

[19] Wang B, Lei Y, Li N. A hybrid prognostics approach for estimating remaining useful life of rolling element bearings. IEEE Transactions on Reliability. 2018;:1–12.

